# Global Rsh-dependent transcription profile of *Brucella suis* during stringent response unravels adaptation to nutrient starvation and cross-talk with other stress responses

**DOI:** 10.1186/1471-2164-14-459

**Published:** 2013-07-08

**Authors:** Nabil Hanna, Safia Ouahrani-Bettache, Kenneth L Drake, L Garry Adams, Stephan Köhler, Alessandra Occhialini

**Affiliations:** 1 Université Montpellier 1, Centre d’études d’agents Pathogènes et Biotechnologies pour la Santé (CPBS), Montpellier, France; 2 CNRS, UMR 5236, CPBS, Montpellier, France; 3 Université Montpellier 2, CPBS, Montpellier, France; 4 Seralogix, Limited Liability Company, Austin, TX, USA; 5 Department of Veterinary Pathobiology, College of Veterinary Medicine and Biomedical Sciences, Texas A&M University, College Station, TX, USA

**Keywords:** Brucella, Stringent response, Transcriptome, Virulence, Methionine biosynthesis, Low oxygen stress, Oxidative stress

## Abstract

**Background:**

In the intracellular pathogen *Brucella* spp., the activation of the stringent response, a global regulatory network providing rapid adaptation to growth-affecting stress conditions such as nutrient deficiency, is essential for replication in the host. A single, bi-functional enzyme Rsh catalyzes synthesis and hydrolysis of the alarmone (p)ppGpp, responsible for differential gene expression under stringent conditions.

**Results:**

cDNA microarray analysis allowed characterization of the transcriptional profiles of the *B. suis* 1330 wild-type and Δ*rsh* mutant in a minimal medium, partially mimicking the nutrient-poor intramacrophagic environment. A total of 379 genes (11.6% of the genome) were differentially expressed in a *rsh*-dependent manner, of which 198 were up-, and 181 were down-regulated. The pleiotropic character of the response was confirmed, as the genes encoded an important number of transcriptional regulators, cell envelope proteins, stress factors, transport systems, and energy metabolism proteins. Virulence genes such as *narG* and *sodC*, respectively encoding respiratory nitrate reductase and superoxide dismutase, were under the positive control of (p)ppGpp, as well as expression of the *cbb3*-type cytochrome *c* oxidase, essential for chronic murine infection. Methionine was the only amino acid whose biosynthesis was absolutely dependent on stringent response in *B. suis*.

**Conclusions:**

The study illustrated the complexity of the processes involved in adaptation to nutrient starvation, and contributed to a better understanding of the correlation between stringent response and *Brucella* virulence. Most interestingly, it clearly indicated (p)ppGpp-dependent cross-talk between at least three stress responses playing a central role in *Brucella* adaptation to the host: nutrient, oxidative, and low-oxygen stress.

## Background

The Gram negative bacterial pathogen *Brucella* is the causative agent of brucellosis, a major zoonotic disease causing abortion and sterility in animals and “Malta fever” in humans. The latter is characterized by an undulant fever and septicemia which may be followed by a subacute or chronic infection [1]. The intracellular survival and replication of *Brucella* is considered the essential trait of virulence where the *Brucella*-containing phagosomes avoid bactericidal mechanisms by evading fusion with degradative lysosomes, a process which is mainly orchestrated by the type IV secretion system VirB [2,3]. During the infection, *Brucella* is able to survive and to adapt to nutrient-poor conditions like those encountered inside the *Brucella*-containing vacuole. A major bacterial strategy to cope with such conditions is the activation of the stringent response, a global regulatory network providing rapid adaptation to a variety of growth-affecting stress conditions [4]. This rapid adaptation is mediated by the accumulation of an alarmone molecule called (p)ppGpp that binds to RNA polymerase, resulting in a large-scale down-regulation of the translation apparatus [5]. The well-studied stringent response is involved in the adaptation to amino acid starvation [6,7], but also to carbon or nitrogen [6,8-10], iron [11], and fatty acid starvation [12]. In *Escherichia coli*, the level of (p)ppGpp is regulated by the enzymes RelA that synthesizes the alarmone following activation by the presence of uncharged tRNAs, and SpoT, a bifunctional enzyme, able to synthesize and hydrolyse (p)ppGpp [6]. The majority of Gram-positive bacteria and the α-Proteobacteria, to which belongs *Brucella* spp., however, possess a single, RelA-SpoT homologue named Rel or Rsh. RelA-SpoT homologues share both conserved (p)ppGpp synthase and hydrolase domains and were demonstrated to be bifunctional in Gram-positive bacteria and in α-Proteobacteria at the examples of *Sinorhizobium meliloti* and *Rhizobium etli*[10,13-15].

Several studies demonstrated essentiality of the stringent response in many infectious processes. An active stringent response is required for the expression of pneumolysin toxin in *Streptococcus pneumoniae*[16], invasiveness and the expression of the type IV secretion system Dot/Icm in *Legionella pneumophila*[17], and mycobacterial long-term survival within macrophages [18] as well as persistence in the murine model [19]. In biofilms, which are related to many chronic infections, nutrient limitation results in a stringent response-dependent antibiotic tolerance and increased antioxydant defenses [20]. In symbiotic α-Proteobacteria such as *S. meliloti* and *R. etli*, the stringent response controls bacterial physiology which is critical to the establishment of a successful symbiosis [15,21]. In the latter, (p)ppGpp-dependent genes have also been identified during active growth in early exponential phase [15].

In *Brucella*, the unique *rsh* gene encodes a protein of 751 amino acids, and homology analysis showed that the synthase and hydrolase residues were conserved, which suggests that Rsh is bifunctional. In a previous study, we demonstrated the role of *rsh* in successful intracellular adaptation [22]. The *rsh* null mutant showed altered morphology, reduced survival in synthetic minimal medium, strong attenuation in cellular and murine models of infection, and lack of induction of *virB* expression [23]. A *rsh* mutant of *Brucella abortus* is also strongly attenuated in the macrophage model of infection and shows a higher sensitivity to NO- and acid pH-mediated bacterial killing, resulting in a lower general stress resistance than the wild-type strain [24]. Such a wide-range regulation necessitates a global gene expression study to elucidate the role of (p)ppGpp in controlling the regulatory processes and networks implicated in the survival and adaptation of brucellae to various environmental conditions. In this study, we determined the transcription profiles of the wild-type and of a *rsh* mutant of *Brucella suis*, a *Brucella* species pathogenic for humans, after stringent response induction in a synthetic minimal medium, partially mimicking conditions encountered by the pathogen within the host cell. Transcription profiling allowed the identification of the global *rsh*-dependent regulatory network in this pathogenic α-Proteobacterium, revealing, amongst others, stringent response control of methionine biosynthesis as well as of nitrate reductase, Cu, Zn superoxide dismutase and *cbb3*-type cytochrome c oxidase. Moreover, the large number of differentially regulated genes was consistent with the pleiotropic effect of the response.

## Results

### Differential gene expression during stringent response in *B. suis*

Expression profiles of *B. suis* 1330 wild-type and the Δ*rsh* mutant were generated using bacteria incubated for 4 h in minimal medium at pH 7.0, conditions known to induce expression of *rsh-*regulated genes in *Brucella* spp. and necessitating the presence of Rsh for growth [23]. Viability of the *rsh* mutant was not affected during this incubation period (not shown). Prior to transcriptional analysis, the deletion of *rsh* in the Δ*rsh* mutant was controlled and confirmed by PCR using primers flanking the mutated region. The whole-genome microarray transcriptional profiling yielded a signal for *rsh* expression also in the Δ*rsh* mutant, as the sequence of the spotted 70-mer oligo specific for *rsh* is located downstream of the deleted region. In addition, RT-qPCR showed that the transcription level of the neighboring gene *pyrE* was not significantly affected, thus confirming that the Δ*rsh* mutation has no polar effect on transcription of genes located downstream.

**Figure 1:**
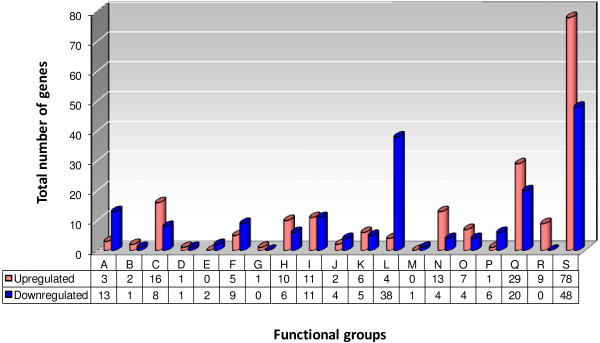
**Stringent response**-**regulated genes of *Brucella suis *1330 have been classified into 19 functional categories.** A total of 379 genes were differentially regulated in minimal medium, representing 11.6% of the *Brucella* genome. “Up-” (red bars) and “Down-regulated” (blue bars) refers to the wild-type situation, in the presence of functional Rsh. **A**: Amino acid metabolism; **B**: Cell Division; **C**: Cell envelope; **D**: Central intermediary metabolism; **E**: Chemotaxis and motility; **F**: Cofactor and carrier biosynthesis; **G**: Detoxification; **H**: DNA/RNA metabolism; **I**: Energy metabolism; **J**: Fatty acid metabolism; **K**: Nitrogen metabolism; **L**: Protein metabolism; **M**: Protein modification and repair; **N**: Regulation; **O**: Stress and adaptation/chaperones/protein folding; **P**: Sugar metabolism; **Q**: Transport systems; **R**: Transposon function; **S**: Unknown function.

Comparative transcriptional analysis between *B. suis* wild-type and the Δ*rsh* mutant revealed the Rsh-dependent differential regulation of 379 genes, which accounts for 11.6% of the genome. 198 of these genes (52%) were up-regulated and 181 genes (48%) were down-regulated by Rsh (Figure 1, and Additional file 1: Table S1).

The differential expression varied between a 5.3-fold Rsh-dependent up-regulation of a gene coding for a protein of unknown function (BR0629; expressed as log2: 2.41), and an 8.4-fold Rsh-dependent down-regulation (*ureB-1*; expressed as log2: -3.06). Differentially expressed genes were grouped into functional categories (Additional file 1: Table S1). Genes were relatively equally distributed over the different functional groups, except for a very strong representation of genes encoding proteins with unknown functions (32%). It appeared, as expected, that in *B. suis*, Rsh controlled a variety of different metabolic pathways through transcriptional regulation of a large number of genes. Consistent with the previously described link between the stringent response and production of (p)ppGpp, a total of 120 of the differentially expressed genes were involved in amino acid, nucleic acid, energy, fatty acid, nitrogen, protein or sugar metabolism. 31% of these metabolism genes were up-regulated and 69% were down-regulated in the presence of a functional *rsh* gene.

#### Genes up-regulated during stringent response

198 (52%) of the differentially expressed genes identified in this study were positively regulated by Rsh, characterized by a significantly higher expression rate in the wild-type than in the Δ*rsh* strain (Additional file 1: Table S1). Among the up-regulated genes, only three (BR0793, BRA0338, BRA0340) were homologous to genes encoding enzymes involved in amino acid metabolism: BRA0338, homologous to genes encoding glutamate decarboxylase and possessing an authentic point mutation in *B. suis*, BRA0340, encoding a glutaminase catalyzing the formation of glutamate from glutamine, and BR0793 encoding O-acetylhomoserine sulfhydrylase, participating in the methionine biosynthesis pathway. Ten genes were implicated in DNA/RNA metabolism, including *himA* (BR0778) encoding the integration host factor (IHF) alpha subunit. As a transcriptional regulator, IHF binds to the promoter of the *virB* operon, participating in control of its expression during the intracellular and vegetative growth in various media [25]. Twelve genes, including *narG* and *narJ* from the respiratory nitrate reductase operon, and *ccoN* and *ccoP* encoding 2 *cbb*_*3*_-type cytochrome *c* oxidase subunits, were involved in energy metabolism. The *cbb*_*3*_-type cytochrome *c* oxidase is a high-oxygen-affinity terminal oxidase and hence allows brucellae to adapt to low oxygen tension. It was identified to be strongly induced under microaerobic conditions *in vitro* and essential for chronic, *B. suis*-mediated murine infection [26,27]. Six genes involved in nitrogen metabolism and belonging to the *ure2*-operon were up-regulated, as well as four genes classified as belonging to protein metabolism, and one gene involved in sugar metabolism. Two genes participating in fatty acid metabolism were also up-regulated, BR1510 encoding an acyl-CoA hydrolase, and *fadD* (BR0289) which encodes an acyl-CoA synthase described to be required for the growth of *Mycobacterium tuberculosis* in a hepatocyte cell line and in mice [28,29] and implicated in sulfolipid production and macrophage adhesion [30]. The gene *sodC*, encoding a Cu, Zn superoxide dismutase participating in detoxification of free oxygen radicals [31], was also up-regulated. In addition, we observed Rsh-dependent up-regulation of two *ftsK* genes encoding cell division proteins. This is consistent with the morphological abnormalities described in our and another previous study on *B. abortus*, where a high proportion of the bacteria were characterized by a branched or unusually swollen phenotype [23,24]. FtsK has been described to couple cell division with the segregation of the chromosome terminus [32].

The stringent response also positively affected stress-related gene expression of a number of heat and cold shock genes and molecular chaperones (*grpE*, *hdeA*, *csp*, *dnaJ*, *usp*). Transcription of *hdeA* (BRA0341) was up-regulated in the presence of *rsh*, and in *B. abortus*, HdeA contributes to acid resistance but is not required for virulence in the Balb/c murine model [33]. Several of the genes up-regulated in a Rsh-dependent manner encode functions related to transcription, with the identification of twelve transcriptional regulators belonging to different families (GntR, RpiR, MerR, LysR, Ros/MucR). GntR5 has been identified as HutC, a transcriptional regulator that exerts two different roles, as it acts as a co-activator of transcription of the *virB* operon, and represses the *hut* genes implicated in the histidine utilization pathway [34]. The regulator MucR has been described as being involved in virulence of *Brucella melitensis* and *B. abortus* in macrophage and murine models of infection [35,36], in lipid A-core and cyclic-β-glucan synthesis in *B. melitensis*[37], and in the successful establishment of symbiosis in *S. meliloti*, including control of exopolysaccharide biosynthesis, necessary for biofilm formation [38,39].

Several other genes encoding factors associated with *Brucella* virulence have been identified as being regulated by (p)ppGpp, which is in agreement with observations made in other bacterial pathogens [17,40,41]. These genes, up-regulated in a Rsh-dependent manner and identified in independent virulence screens [42] (the latter as review) include those involved in cell envelope formation (*omp19*, *wbpL*, *lpsA*, *amiC*, *wbdA*), in DNA/RNA metabolism (*mutM* and *pyrB*), in stress response (*csp*), in transport/secretion systems (*virB5* and *dppA*), and one gene encoding a protein of unknown function. The roles of the *cbb*_*3*_-type cytochrome *c* oxidase (encoded by the genes of the operon *cco*) and of the Cu, Zn superoxide dismutase (encoded by *sodC*) were described elsewhere [26,27]. Table 1 lists these 15 genes positively regulated by (p)ppGpp and participating in *Brucella* virulence.

**Table 1:** **Genes identified as being positively regulated by Rsh during stringent response, and previously described as essential for virulence of *Brucella *spp. in *in vitro *and/or *in vivo *models of infection**

***B. suis*Gene ID**	***B. melitensis*Gene ID**	**Gene**	**Putative or assessed function**	**References**
BR0511	BMEI1426	*wbpL*	O-chain biosynthesis	[35,42]
BR0615	BMEI1326	*lpsA*	Putative glycosyltransferase	[35,42]
BR0915	BMEI1056	*amiC*	Cell-wall hydrolysis	[42]
BR0982	BMEI0997	*wbdA*	O-chain biosynthesis	[35,42]
BR1930	BMEI0135	*omp19*	Lipoprotein	[42]
BRA0703	BMEII0581	*sodC*	Cu/Zn super oxide dismutase	[42,52]
BR2183	BMEI1946	*mutM*	Repair from mutagenesis by alkylating agents	[42]
BRA0599	BMEII0670	*pyrB*	Pyrimidines biosynthesis	[42]
BR0363, BR0360	BMEI1564, BMEI1566	*ccoN, P*	cytochrome *c* oxidase, *cbb3*-type	[26,27]
BR0569	BMEI1364	*mucR*	Transcriptional regulator	[35]
BR1514	BMEI0498	*csp*	Cold shock protein	[35]
BRA0065	BMEII0029	*virB5*	Macromolecule secretion	[42]
BRA1012	BMEI0433	*dppA*	Dipeptide uptake	[42]
BR0049	BMEI1894	*-*	Conserved hypothetical protein	[35]

Genes belonging to same functional groups are listed together.

#### Genes down-regulated during stringent response

In parallel, transcriptome analysis revealed 181 down-regulated genes (Additional file 1: Table S1). The gene most strongly down-regulated in the presence of Rsh was *ureB1* encoding the urease beta-subunit, involved in nitrogen metabolism and forming part of the *ure*-1 operon. The other four genes identified as belonging to the *ure*-1 operon (*ureC, ureD1, ureE1*, and *ureG1*) were also repressed. The hallmark of the stringent response is the (p)ppGpp-dependent down-regulation of transcription of the genes encoding ribosomal proteins. In fact, in our transcriptome analysis, all the genes encoding ribosomal proteins identified in this transcriptome (n = 29) were down-regulated. Among the genes coding for transcriptional regulators, we identified three genes (BR1187, BR1378 and BR0872) belonging to the MerR, AspB, and ExoR family, respectively, as being down-regulated, the latter being described as repressing exopolysaccharide production. Interestingly, two genes of the flagella clusters, identified as *flaF* and *fliG*, were also affected. Flagella can be detected under certain culture conditions in *Brucella*, but their biological function remains yet unknown [43]. Fatty acid and glycerophospholipid biosynthesis were most likely reduced during stringent response, as we identified four genes involved in these processes being down-regulated in the presence of Rsh. Similarly, metabolism of sugars and glycolysis were apparently diminished, in agreement with a general reduction of metabolic activities. We also observed the down-regulation of the gene encoding GlnE, responsible for adenylylation of glutamine synthetase (GS). Reduced adenylylation increases GS activity, and the synthetized glutamine plays a central role in the biosynthesis of amino acids, purines, and pyrimidines.

In addition, we found a wide range of genes encoding ORFs with unknown functions to be differentially regulated during stringent response.

### Validation of the microarray expression data by RT-qPCR analysis

**Figure 2:**
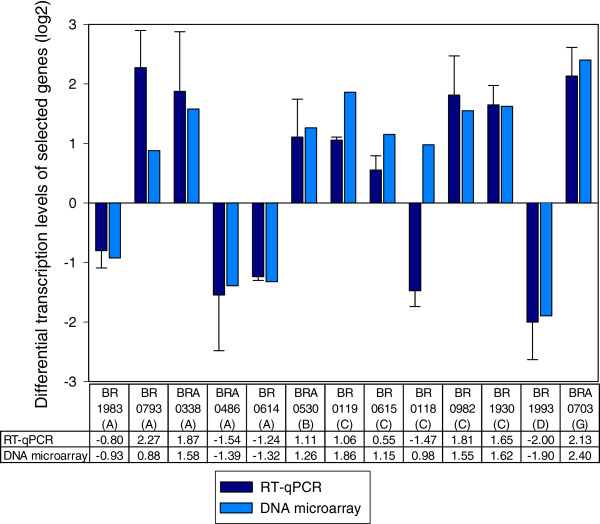
**Comparison of microarray analysis to real**-**time PCR revealed 78% of true positives.** Log2-values of fold-changes of differentially expressed genes under stringent conditions are shown for normalized microarray data and RT-qPCR of 13 ORFs (out of 40 total; see also Additional file 2: Figure S1) representing the different functional groups: A- Amino acid metabolism; B- Cell Division; C- Cell envelope; D- Central intermediary metabolism; G- Detoxification. Expression of 78% of all chosen ORFs (including those presented in Additional file 2: Figure S1) was consistent for both methods, with a log2(x) > 0.66 or log2(1/x) < −0.66 (representing a plain fold-change > 1.5). For the remaining 22%, the fold-change was inferior to log2(x) = 0.66 or superior to log2(1/x) = −0.66 (representing a fold change < 1.5) or not consistent with microarray analysis. Relative differences of transcription levels between *B. suis* wild-type and the Δ*rsh* mutant were determined as 2^-ΔΔCt^ values, as described in Methods.

RT-qPCR was used to validate the expression trends of selected genes identified as being differentially expressed by microarray analysis. Using the same total RNA preparations as for the microarray hybridizations, expression of selected genes, representing the previously defined different functional groups, was analyzed by RT-qPCR. Gene BR1035 of unknown function was used as internal reference for normalization, as its expression rate was constant for both strains in the experiments. The expression ratios obtained from RT-qPCR and microarrays were plotted for comparison (Figure 2, and Additional file 2: Figure S1).

We investigated a total of 40 genes representing most of the previously defined functional groups. Using RT-qPCR, thirty one (78%) of the tested genes were found to be differentially expressed between wild-type and *rsh*-mutant, when applying a cutoff value of log2(x) > 0.66 or log2(1/x) < −0.66 (i.e. a plain fold change > 1.5), and each of these genes was regulated in the same direction (up or down), according to both RT-qPCR and the microarray study. Four other genes were identified as being regulated in the same direction (up or down) for both RT-qPCR and microarray analysis, but the rates of differential expression were not significant for RT-qPCR. Up-or down-regulation opposite to the direction of regulation obtained by the microarray study was observed by RT-qPCR for the five remaining genes, out of the total of 40. Therefore, the overall correlation between the expression levels obtained by microarrays and those obtained by RT-qPCR was good. Altogether, these results confirmed that *rsh*-dependent differential gene expression evidenced by oligonucleotide microarray analysis could be reproduced by RT-qPCR.

Nitrate reductase, *cbb3*-type cytochrome *c* oxidase, and Cu, Zn superoxide dismutase (SOD) of *B. suis* are under the positive control of (p)ppGpp.

Induction of the *nar* operon by (p)ppGpp, as observed by microarray analysis and RT-qPCR (Additional files 1: Table S1 and 2: Figure S1), was confirmed biochemically by measurement of the enzymatic activity of the nitrate reductase operon. After a 4 h-induction of the bacteria in GMM, the NO_2_^−^ production was measured in the bacterial culture supernatants. Consistent with the microarray and RT-qPCR results, significant production of NO_2_^−^ was observed in the *B. suis* wild-type strain with a mean nitrite concentration of 698.5 ± 6.5 μM, as compared to its Δ*rsh* isogenic mutant with a strongly reduced nitrite concentration of only 40.2 ± 6.4 μM. A Δ*narG* mutant, deficient in nitrite production, was used as a negative control [44]. In the same functional group of energy metabolism, two genes encoding subunits of the *cbb3*-type cytochrome *c* oxidase were also induced (Additional files 1: Table S1 and 2: Figure S1).

**Figure 3:**
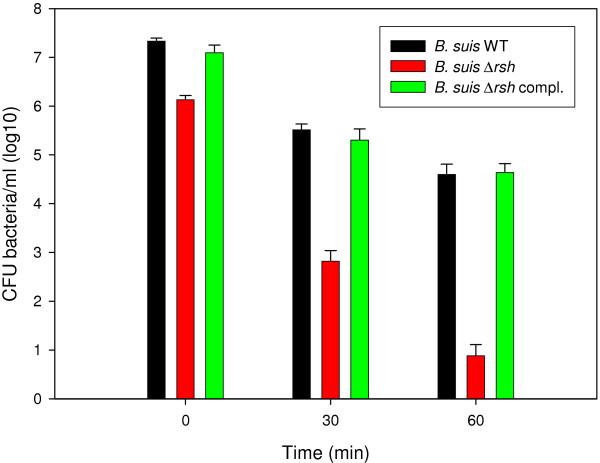
**The Δ*rsh *mutant is highly sensitive to exposure to O**_**2 **_^**-**^**.** Determination of the concentrations of viable *B. suis* wild-type (black bars), Δ*rsh* mutant (red bars), and complemented *rsh* mutant (green bars) following 30 and 60 min of exposure to O_2 _^-^ generated by the xanthine oxidase reaction, as described in Methods.

Both microarray and RT-qPCR results also revealed that induction of *sodC*, encoding Cu, Zn superoxide dismutase, was *rsh*-dependent (Figure 2, Additional file 1: Table S1). Production of exogenous O_2 _^-^ was artificially induced by the xanthine oxydase reaction, where xanthine is converted to urate, generating O_2 _^-^. The number of surviving bacteria in the cell suspension was determined at specific time points thereafter. Despite lower initial survival of brucellae in the preculture due to pleiotropic effects of *rsh* mutation, there was a clear correlation between *rsh* expression and resistance to O_2_^-^ radicals: the net difference in survival between the wild-type and the Δ*rsh* strain was 50-fold at 30 min and 500-fold at 1 h (Figure 3). The higher sensitivity of the *rsh* mutant to O_2_^-^ was completely abolished following complementation with the intact gene and survival was not significantly different from that of the wild-type strain (Figure 3).

### Growth of a *rsh* null mutant in minimal medium requires methionine, present in the *Brucella*-containing vacuole of macrophages

**Figure 4:**
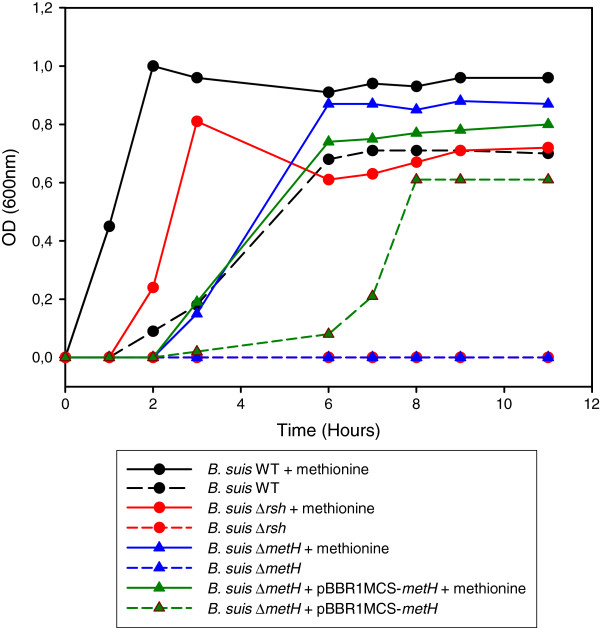
**Growth of the Δ*rsh *mutant in minimal medium requires addition of methionine only. ***B. suis* 1330 wild-type (black), *B. suis* strains Δ*rsh* (red), Δ*metH* (blue) and Δ*metH* complemented with the *metH* gene on plasmid pBBR1-MCS (green), were grown in TS medium overnight, washed twice, and diluted 1/500 in GMM in the absence (dashed lines) or presence (solid lines) of methionine. Bacterial growth was measured at λ 600 nm. Both Δ*rsh* and Δ*metH* mutants were unable to grow in the absence of methionine and both graphs superpose as a “base line”. +met: addition of exogenous methionine; +pBBR1-MCS-metH: complementation plasmid for Δ*metH* mutant.

Lack of stringent response in a Δ*rsh* mutant resulted in lack of growth in GMM, which was restored in the complemented strain [23]. In order to characterize the nutritional requirements of a Δ*rsh* mutant under these growth conditions, we analyzed the amino acid requirements of the *rsh* null mutant in GMM. Each of the 20 culture tubes per *B. suis* wild-type and Δ*rsh* strain, respectively, contained all but one of the 20 amino acids. Control cultures in medium lacking all amino acids and in medium containing all 20 amino acids (not shown), as well as a Δ*metH* mutant and its complemented form, were also included. The Δ*metH* mutant (BR0188) was unable to synthesize 5-methyltetrahydrofolate-homocysteine methyltransferase, the last enzyme in the methionine biosynthesis pathway, transforming L-homocysteine into L-methionine. Results showed that the *rsh* mutant did not grow in medium lacking only methionine (Figure 4). As expected, the Δ*metH* mutant behaved the same way. Growth was restored upon the addition of methionine, indicating that only the methionine biosynthesis pathway was affected in the Δ*rsh* mutant able to synthesize all other amino acids under nutrient starvation (Figure 4). Addition of exogenous methionine to the wild-type and to the complemented Δ*metH* mutant favoured earlier growth of the strains as compared to growth rates in GMM lacking methionine.

**Figure 5:**
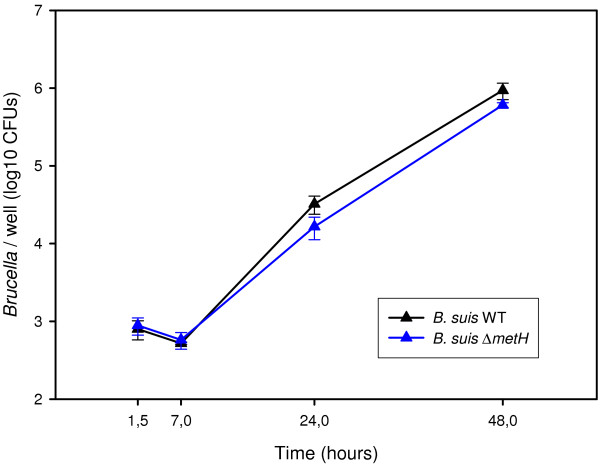
**Intracellular growth of *B. suis *does not require bacterial methionine synthesis.** Murine J774 macrophage-like cells were infected with *B. suis* wild-type (black) and the Δ*metH* mutant (blue). The experiments were performed three times in triplicate each. Data are presented as means ± standard deviations of one representative experiment.

In order to determine if the lack of capacity to synthesize methionine could also explain the inability of the Δ*rsh* mutant to replicate in the macrophage model of infection [22], the above-described *metH* mutant was used in the infection experiments. Interestingly, the Δ*metH* mutant was not attenuated in the J774 murine macrophage model of infection (Figure 5), leading to the conclusion that this amino acid was available in the *Brucella*-containing vacuoles and that the intracellular attenuation of the *rsh* mutant was not due to amino acid starvation.

**Figure 6:**
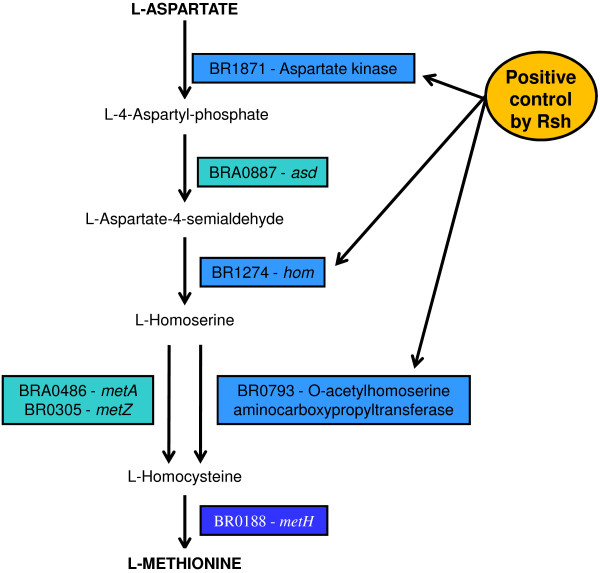
**Three genes of the methionine biosynthesis pathway were positively regulated by (p)ppGpp in *B. suis*.** The different genes encoding the enzymes involved in the pathway from L-Aspartate to L-Methionine are represented. Gene BR0793 was identified by microarray analysis and RT-qPCR, genes BR1871 and BR1274 by RT-qPCR.

Microarrays and RT-qPCR data revealed that *metH* was not regulated in a (p)ppGpp-dependent manner under stringent conditions, in contrast to *metA* (BRA0486) which was down-regulated and to the gene BR0793 which was up-regulated in the presence of Rsh (Figure 2, Additional file 1: Table S1). Mutants respectively carrying the inactivated genes *metA*, *metZ*, or BR0793 encoding O-acetylhomoserine sulfhydrylase, were characterized by reduced growth in GMM (not shown). This result was consistent with the known general methionine biosynthesis pathway composed of two parallel branches, one including *metA* and *metZ* and the other BR0793. RT-qPCR was therefore employed to systematically assess an eventual link between *rsh* and the expression of genes located further “upstream” on the methionine biosynthesis pathway (Figure 6).

The transcripts encoding aspartate kinase (BR1871) with log2(x) of the differential expression ratio wild-type*/rsh* = 0.89 ± 0.15, and homoserine dehydrogenase (*hom*; BR1274) with log2(x) of the differential expression ratio wild-type/*rsh* = 0.83 ± 0.51 were found to be significantly less abundant in the *rsh* mutant than in the wild-type strain. These data indicated that in addition to positively regulating the transcription levels of O-acetylhomoserine sulfhydrylase (BR0793), Rsh was also involved in the positive regulation of the two genes BR1274 and BR1871 encoding proteins further upstream in the central part of the methionine biosynthesis pathway, between the L-aspartate and the L-homoserine intermediate product (Figure 6).

## Discussion

The stringent response reflects the adaptation of a bacterium to nutrient stress via a complex differential gene expression pattern, affecting a large number of structural and regulatory target genes and resulting in a pleiotropic phenotype. In an earlier study, some of us showed that *rsh* deletion mutants were characterized by altered morphology, lack of expression of *virB*, and reduced survival in cellular and murine models of infection [23]. In this study, we investigated the global expression profile of the stringent response using a DNA microarray approach with the aim to characterize the (p)ppGpp-dependent regulatory network, and we also focused on methionine biosynthesis, the sole amino acid whose biosynthesis pathway was controlled by stringent response in *B. suis*.

A comparison of the wild-type and *rsh* mutant transcriptomes showed that approximately 12% of the *B. suis* genome were under the control of Rsh, of which 52% were up-regulated and 48% were down-regulated. The differentially transcribed genes were classified into 19 functional categories.

A key element in the establishment of *Brucella* infection is the ability of the bacterium to resist to acid pH and nutrient deprivation within the macrophage host cells, at least in the early phase of infection. Both signals are essential for a strong induction of the T4SS in *B. suis*, although RNA blot experiments with a *virB5*-probe reveal some *virB*-expression in minimal medium at pH 7 [45], corresponding to our experimental conditions in this transcriptome study. Our transcriptome study showed that *virB*-expression was Rsh-dependent, which is in accordance with previous work of some of us [23]. On a fine-tune level, the gene encoding the alpha-subunit of the Integration Host Factor (IHF) and *hutC*, which both control transcription of the *virB* operon via specific promoter binding sites, were also up-regulated under nutrient stress conditions. In *E. coli*, it has been described that expression of IHF is induced under stringent response conditions and in early-stationary phase [46,47]. The Rsh-dependent up-regulation of IHF-expression in *Brucella* confirmed the regulation pathway previously suggested: *Brucella* senses nutrient starvation via Rsh, resulting in (p)ppGpp production, which will then increase transcription of IHF, affecting the activity of the *Brucella virB* promoter [48]. However, the fact that overexpression of the *virB* operon in a *B. suis* Δ*rsh* background did not restore the parental phenotype by itself (data not shown) is an additional indication that the stringent response has a pleiotropic effect on virulence factor expression. Among transcriptional regulators participating in *Brucella* virulence, expression of MucR was shown to be Rsh-dependent. Studies on a *mucR*-mutant of *B. melitensis* suggest that MucR regulates genes involved in nitrogen metabolism and stress response [49], as well as in lipid A-core and cyclic-β-glucan synthesis of this pathogen [37]. In this context it is important to mention that recent work on the transcriptome analysis of stringent response in the α-proteobacterium and plant symbiont *R. etli* reported the observation that Rsh positively controls expression of key regulators for survival during heat and oxidative stress [15]. Remarkably, about 6% of the (p)ppGpp-regulated genes in *B. suis* are transcriptional regulators of, most-often, uncharacterized function.

Several consecutive genes, potentially involved in resistance to acid pH, were also induced by Rsh: genes encoding the glutamate decarboxylase A (BRA0338), the glutaminase (BRA0340), and *hdeA* (BRA0341). Glutamate decarboxylase, which is involved in resistance of *Brucella microti* to pH 2.5 [50] is not functional in *B. suis*. This overexpression could be the remnant of a function that has been lost during the evolution of *Brucella*. However, this gene potentially forms an operon with *hdeA* (unpublished results), which plays a role in the resistance to acid stress in *B. abortus*[33]. These results suggest that Rsh may prepare the bacteria to respond to a possible acid stress subsequent to nutrient deficiency.

Experimental evidence indicates that production of reactive oxygen intermediates (ROIs) represents one of the primary antimicrobial mechanisms, and ROI formation has been described during macrophage infection by *Brucella*[51]. Among the genes positively regulated by Rsh that contribute to the virulence of *Brucella*, we identified *sodC* encoding the Cu-Zn superoxide dismutase. A *sodC* mutant of *B. abortus* was more sensitive to killing by O_2_^−^ than the wild-type, and exhibited an increased susceptibility to killing in cultured macrophages and in a murine model of infection [52]. We suggest that the lack of up-regulation of this protective gene might participate in the attenuation previously described for the Δ*rsh* mutants of *Brucella*[22,23]. In addition, it has been reported recently for biofilm-forming *P. aeruginosa* that an active stringent response increased not only antibiotic tolerance, but also antioxydant defenses by increasing production of active catalase and SOD against endogenous oxydant production [20]. This indicated at least a partial conservation of stringent response-dependent adaptation strategies of different bacterial pathogens to various stress conditions encountered during chronic infection or biofilm formation.

One of the hallmarks of stringent response is the down-regulation of protein synthesis, together with induction of amino acid biosynthesis pathways. Based on the transcriptome results obtained for the well-studied stringent response of *E. coli*[7,53] and on earlier reports [54], a major event of the stringent response consists in the repression of the translation apparatus, including ribosomal proteins. As expected, the transcriptome data of our study clearly demonstrated that the regulation of the translation apparatus is (p)ppGpp-dependent, as we identified 29 genes coding for ribosomal proteins that were down-regulated. The microarrays used in this study lack the genes of the rRNA operons of *Brucella*, explaining why these genes were not also identified as being down-regulated in the wild-type strain during stringent response. Regarding induction of amino acid biosynthesis pathways, it has been described that these pathways differ, depending on the bacterial species studied. In *E. coli*, the stringent response positively controls the biosynthesis of the branched-chain amino acids, glutamine/glutamate, histidine, lysine, methionine and threonine [54]. In *B. subtilis*, proteome and transcriptome analysis has shown induction of enzymes involved in branched-chain amino acid biosynthesis during stringent response [55]. The assessment of the amino acid biosynthesis pathways positively regulated by Rsh during the stringent response in *B. suis* revealed that only methionine biosynthesis was controlled by (p)ppGpp. As we previously put forward the hypothesis of a nutrient-poor environment during macrophage infection, based on the observation of the attenuation of several mutants affected in amino acid and nucleotide biosynthesis pathways [22,56], we verified the possibility that Rsh-dependent methionine synthesis might be crucial during infection. We therefore constructed a Δ*metH* allelic exchange mutant of *B. suis*, which was auxotrophic. Intracellular growth of the Δ*metH* mutant, however, was not impaired, indicating that the lack of methionine biosynthesis in the Δ*rsh* mutant did not contribute to its attenuation and that methionine was available in the *Brucella*-containing vacuole. Our results are in contrast to those described by Lestrate *et al.*[57], who observed attenuation of a *B. melitensis metH* transposon mutant *in vitro* and *in vivo*, but, surprisingly, in the absence of any auxotrophic phenotype in minimal medium. In addition, genome sequencing of *B. melitensis* has since revealed that a gene encoding an alternative enzyme functionally replacing MetH is not present [58]. Interestingly, in the α-proteobacterium and plant symbiont *R. etli*, (p)ppGpp-dependent upregulation of any amino acid biosynthesis pathways during stationary phase could not be evidenced. Rather, amino acid biosynthesis was down-regulated in a (p)ppGpp-independent manner under these growth conditions [15]. It therefore appears that induction of amino acid biosynthesis during stringent response is not a key feature in α-proteobacteria.

The *nar* operon, which encodes the respiratory nitrate reductase in *Brucella*, was also induced under stringent conditions, as evidenced by the microarray analysis and by dosage of the nitrites produced by the nitrate reductase. *Nar*-induction was shown by the Rsh-dependent, positive control of the genes *narG, narJ, and narK*, involved in the first step of denitrification consisting in the reduction of nitrate to nitrite, and in nitrite extrusion towards the periplasm [59,60]. This denitrification pathway may allow *Brucella* to survive under low oxygen tension, using nitrogen oxides as terminal electron acceptors [61]. Denitrification can also be used by brucellae to detoxify NO produced by activated murine macrophages during infection, and part of this denitrification island is important for virulence of *B. suis in vivo*[44]. Recent proteomic and gene fusion studies with *B. suis* evidenced the induction of the *nar* operon under microaerobic conditions [61]. In *E. coli*, expression of the *narGHJI* operon is induced by low oxygen tension and by the presence of nitrate [62], which is imported by NarK, also responsible for nitrite export [63]. One might speculate that our observations made in GMM broth were due to low-oxygen exposure of the strains, but this appears unlikely as pre-cultures were diluted in minimal medium under vigorous shaking for 4 hours. In *M. tuberculosis*, oxygen concentration-independent expression of *nar* has been described [64], and nitrate reductase component genes *narH* and *narI* have been identified as being under the positive control of Rel_Mtb_ (Rsh) under comparable nutrient starvation culture conditions [19]. It is therefore conceivable that the *Brucella* nitrate reductase is expressed in a stringent response-dependent manner under aerobic conditions, and further induced under hypoxia. In analogy to the observations made for the *nar*-operon, genes encoding *cbb3*-type cytochrome *c* oxidase were also observed being under the positive control of Rsh in *B. suis*, despite normal oxygenation. We hypothesize that stringent response, mediating adaptation of the pathogen to nutrient stress, therefore also represents a first step of potential adaptation to successive reduction of oxygen concentrations encountered by the pathogen in the host cells, the target organs and granulomes or abcesses.

The gene *sodC*, encoding Cu, Zn superoxide dismutase, was also positively regulated by the product of Rsh. The mutant was significantly more sensitive to O_2_^-^ radicals then the wild-type *in vitro*, already at short times of treatment. It has been described previously that a *sodC* mutant of *B. abortus* exhibited much greater susceptibility to killing by O_2_^-^ than the parental strain [52]. The same mutant was also much more sensitive to killing in cultured macrophages, as well as in the murine model of *in vivo* infection, due to its inability to detoxify the O_2 _^-^ generated by the respiratory burst of the phagocytes [52]. In addition to the previous observation of some of us that *virB* expression is Rsh-dependent [23], we therefore have now validated by a biological survival assay a second *Brucella* gene product whose activity was necessary for intramacrophagic and intramurine replication and whose expression was controlled by Rsh during starvation. In *B. suis*, stringent response therefore also participated in protection from oxidative stress. A similar observation has been reported lately for *Pseudomonas aeruginosa*, where stringent response mediates increase of antioxidant defenses [20].

Interestingly, both urease operons of *B. suis* were regulated during stringent response in GMM: The *ure-1* operon, responsible for the urease activity observed in most species [65,66], was less expressed in the (p)ppGpp-producing wild-type strain than in the mutant, whereas *ure-2* was more expressed under these conditions. In rich broth, i.e. under non-stringent conditions, urease activity, correlating with expression, has been described to be at its maximum in the absence of ammonium chloride, and enzymatic activity decreases with increasing ammonium concentrations [66]. Interestingly, a similar negative regulation of urease expression during stringent response has been described in *Corynebacterium glutamicum*[67]. The functions of the *ure-2* cluster have been unraveled only recently: it is composed of genes encoding a urea and a nickel transport system within a single operon [68]. Our results are in agreement with those published by Rossetti *et al.*, comparing the transcriptional profiles of *B. abortus* under logarithmic and stationary growth phase conditions: the *ure-2* operon is induced during stationary phase, known to be triggered by stringent response [69].

The observation that *flaF* and *fliG*, belonging to flagellar loci I and loci III, respectively, were down-regulated, suggested that the flagellar apparatus of *Brucella* was repressed under stringent conditions. This result is in agreement with the *E. coli* transcription profile of the stringent response, where the expression of the *flhDC* flagella master regulator is rapidly down-regulated [70]. Hence, bacteria appear to shut down transcription of the flagellar cascade under starvation. This strategy makes physiological sense, as it would avoid the expending scarce energy resources for one of the bacteria’s largest macromolecular complexes. In brucellae, the biological function of the flagellar components has not been determined yet, especially in the context of host cell infection. Very recently, down-regulation of flagellar genes expression by the transcriptional regulator MucR has been described in *B. melitensis*[37]. Our observation that MucR is positively regulated during stringent response in *B. suis*, whereas genes encoding the flagellar apparatus are down-regulated under the same conditions, indicates that such a link also exists in *B. suis* and, more generally, that (p)ppGpp is located high up in the hierarchy of gene regulation in *Brucella* spp.

Altogether, the transcription analysis of the stringent response in the facultatively intracellular pathogen *B. suis* confirmed the pleiotropic character of the (p)ppGpp-mediated adaptation to poor nutrient conditions. Several of the genes identified as being under (p)ppGpp control have also been described previously as being essential for the virulence of the pathogen [42], establishing a link between stringent response and virulence. Among these, five genes encoded proteins involved in cell envelope biosynthesis, of which four are essential for intramacrophagic replication of *Brucella*[42]. In addition, four outer membrane proteins (Omps) encoded by the genes BR0119, BR0971 (both putative Omps), BRA0423 (“Omp31-2”), and BR1622 (“Omp31-1”), all under the positive control of (p)ppGpp during stringent response, have also been identified previously as being up-regulated in intramacrophagic *B. suis* at 48 h post infection [71]. At this rather late stage of intracellular infection, *B. suis* shows a high level of replication, and these four genes/proteins were described as being up-regulated in both studies, indicating the potential importance of these Omps throughout the various stages of intramacrophagic infection.

Last not least, the function of 33% of the genes encoding Rsh-dependent transcripts remains unknown. This illustrates the complexity of the processes involved in adaptation to nutrient starvation while bearing in mind that knowledge of a substantial part of the *Brucella* genome is still limited. In this context, it is also worthwhile to mention that in *E. coli*, stringent response induces the alternative, stationary-phase sigma factor RpoS [7,53]. In brucellae and other α-proteobacterial species, however, a functional analogue of RpoS was unknown. Only very recently, an intact general stress response system (GSR), including genes encoding the regulator *phyR* and the alternative sigma factor *rpoE1*, has been described for *B. abortus*[72]. The transcriptome analysis of the stringent response in *B. suis* described here did not give any indication that stringent response might directly affect expression of these two GSR-related genes. Either stringent response controls another, yet uncharacterized alternative sigma factor in *Brucella*, or binding of (p)ppGpp to the RNA polymerase core affects competition between sigma factors in favor of an alternative sigma factor, which could then be RpoE1, increasing specific expression of stress-related genes.

## Conclusions

Rsh, the enzyme controlling stringent response by the synthesis or hydrolysis of (p)ppGpp, affects expression of more than 10% of the genes of *B. suis* under nutrient starvation conditions. (p)ppGpp, as a regulator of gene expression, is thereby located high up in the hierarchy of gene regulation in *Brucella* spp., as we identified 17 transcriptional regulators under the control of Rsh. Expression of *virB*, the secretion system which plays a central role in *Brucella* virulence, is controlled by two transcriptional regulators which have been shown to be Rsh-dependent. Our novel data from the first stringent response transcriptome analysis of an α-proteobacterial pathogen confirm that expression of additional virulence genes is also controlled by Rsh, as 14 other genes previously identified as essential for *Brucella* virulence are up-regulated by Rsh under nutrient starvation. This fact emphasizes the importance of stringent response in adaptation of this pathogen to the host, and yields additional explanation for our previous observations that Rsh is essential for intramacrophagic and intramurine replication.

In contrast to the work on stringent response published for other bacteria, where the biosynthesis pathways of several amino acids, respectively, are under positive (p)ppGpp control, only methionine biosynthesis is concerned in *B. suis*. As this amino acid is obviously not lacking in the *Brucella*-containing vacuole of the macrophage, we conclude that stringent response, triggered by nutrient starvation in this compartment, is crucial for the induction of a set of virulence genes, but not for the induction of the biosynthesis pathways needed to provide the lacking amino acids.

The transcriptome analysis of the stringent response in *B. suis* lead to the observation that the *rsh* mutant had a lower general stress resistance than the wild-type strain, allowing the hypothesis that stringent response and adaptation to nutrient stress may in fact trigger cross-talk with other general stress responses in *Brucella*, thereby preparing the pathogen to different stress conditions possibly encountered simultaneously or soon after. In the life cycle of *B. suis* within the host, starvation inside the macrophage cell may be the first major stress encountered. According to the transcriptome data discussed above, such cross-talk may take place between at least three stress responses playing a central role in *Brucella* adaptation to host: nutrient stress, oxidative stress and low-oxygen stress.

## Methods

### Bacterial strains and media

The *Brucella* reference strains used in this study were *B. suis* 1330 (ATCC 23444) and *B. melitensis* 16 M (ATCC 23456). Ultra-competent *E. coli* DH5α (Invitrogen) were used for cloning and plasmid production. *Brucella* and *E. coli* strains were grown in Tryptic Soy (TS) and Luria Bertani (LB) broth (Invitrogen, Carlsbad, CA, U.S.A.), respectively. For strains carrying resistance genes, kanamycin, ampicillin, and chloramphenicol were used at a final concentration of 50 μg/ml each. *B. suis* stringent response assays were performed as follows: a stationary-phase overnight culture obtained in TS was washed once in phosphate-buffered saline (PBS) prior to a 1:5 dilution in Gerhardt’s Modified Minimal Medium (GMM) adjusted to pH 7.0 [73], followed by incubation at 37°C with shaking for 4 h. Concentrations of live bacteria ml^-1^ were determined prior and after induction by CFU counting after plating of serial dilutions onto TS agar, revealing starting concentrations of approximately 10^9^ viable bacteria ml^-1^ for both strains, and almost identical concentrations at the end of the experiment. Under these conditions, both strains remained fully viable but did not start active growth.

### Growth assays

For the identification of amino acids essential for Δ*rsh* growth in GMM, *B. suis* cultures were grown overnight at 37°C in TS broth. Bacteria were collected by centrifugation at 13,000 rpm for 5 min, washed twice with 9% NaCl, diluted 1:500 and used to inoculate twenty 15-mL tubes containing 3 mL of GMM, where glutamate was replaced by ammonium sulfate, and all natural amino acids added at a concentration of 1 mM each (Sigma), except one. The method was systematically applied to each of the 20 amino acids.

### Isolation and labeling of *B. suis* genomic DNA

Genomic DNA was isolated from *B. suis* culture using Qiagen DNeasy blood and tissue kit and labeled by direct incorporation of Cy-5-dCTP fluorescent dye (Amersham) using the BioPrime DNA labeling system kit (Invitrogen) that contains random primers (octamers) and Klenow fragment. For a 50-μL reaction mixture, 2 μg of genomic DNA template in 23 μL of sterile water were heated at 95°C for 10 min, combined with 20 μl of 2.5X random primers solution, heated again at 95°C for 5 min, and chilled on ice. Remaining components were added to the following final concentrations: 0.12 mM dATP, dGTP, and dTTP; 0.06 mM dCTP; 0.02 mM Cy5-dCTP; 1 mM Tris–HCl (pH 8.0); 0.1 mM EDTA; and 40 units of Klenow fragment. The solution was incubated at 37°C for 2 h before the reaction was stopped by adding EDTA (pH 8.0) to a final concentration of 45 mM. The fluorescence-labeled DNA was purified using the CyScribe GFX purification kit (Amersham Biosciences) and eluted in 1 mM Tris pH 8.0 and kept in the dark at 4°C.

### Isolation of total RNA from *B. suis*

Expression profiles of *B. suis* 1330 wild-type and the Δ*rsh* mutant were generated using 9 mL of bacteria incubated in GMM containing ammonium sulfate instead of glutamate, to ensure total amino acid starvation, at pH 7.0 for 4 h, conditions that are known to induce expression of *rsh-*regulated genes [23]. For each strain, four independent RNA preparations from four independent cultures were used. RNA extraction was performed with the RNeasy mini kit from Qiagen according to the manufacturer’s instructions, with a modified lysis step.

Briefly, after addition of ethanol/phenol solution (9:1) to the cultures, the bacteria were recovered by centrifugation. The bacterial pellet was suspended in TE buffer-lysozyme solution (Invitrogen). After 5 min of incubation, the lysate was mixed with 10% SDS and proteinase K, and incubated for 10 min at 25°C. Buffer RLT with beta-mercaptoethanol was added to the sample followed by centrifugation. The supernatant containing the RNA was recovered and transferred to RNeasy mini spin column. RNA samples were treated with RNAse-free DNase I (Ambion) according to the manufacturer’s instructions. RNA concentration was determined at λ 260 nm using the NanoDrop ND-1000, and quality was evaluated using a Nano-Chip on an Agilent 2100 Bioanalyzer.

### Microarray construction

The pattern of the whole-genome oligo arrays was based on the sequenced genome of *B. melitensis*. The 70-base nucleotides representing 3,227 ORFs plus unique sequences from *B. abortus* and *B. suis* were designed by Sigma Genosys [69]. rRNAs-genes were not represented on the microarrays. Oligonucleotides were suspended in 3× SSC (Ambion) at a final concentration of 40 μM prior to robotic arraying in quadruplicates onto ultraGAPS coated glass slides (Corning) using a spotarray 72 microarray printer (Perkin Elmer). Printed slides were steamed, UV cross-linked and stored in desiccators until use.

### Probe labeling and microarray slide hybridization

The RNAs were converted to cDNA and labeled with Cy3 fluorescent dyes (Amersham Biosciences), using a two-step protocol as previously described [74,75]. Briefly, total RNA (30 μg) was first converted to cDNAs with incorporation of a chemically reactive nucleotide analog (amino allyl-dUTP) using Superscript III reverse transcriptase and random hexamers (Invitrogen). This cDNA is then “post labeled” with the reactive forms of fluoroLink Cy3-NHS esters (Amersham), which bind to the modified nucleotides. The labeled cDNAs were then combined with 0.5 μg of labeled gDNA to a final volume of 35 μL. Samples were heated at 95°C for 5 min and then kept at 45°C until hybridization, when 35 μL of 2× formamide-based hybridization buffer (50% formamide; 10× SSC; 0.2% SDS) were added to each sample. Samples were then well-mixed and applied to custom 3.2 K *Brucella* oligo-arrays. Prior to hybridization, oligo-arrays were pretreated by washing in 0.2% SDS, followed by 3 washes in distilled water, and immersed in pre-hybridization buffer (5 × SSC, 0.1% SDS; 1% BSA in 100 ml of water) at 45°C for at least 45 min. Immediately before hybridization, the slides were washed 4 × in distilled water, dipped in 100% isopropanol for 10 sec and dried by centrifugation at 1,000 × *g* for 2 min.

Four slides for each condition (i.e. *B. suis* 1330 wild-type and the *Δrsh* mutant) were hybridized at 45°C for ~ 20 h in a dark, humid chamber (Corning) and then washed for 10 min at 45°C with low stringency buffer (1× SSC, 0.2% SDS), followed by two 5-min washes in a higher stringency buffer (0.1 × SSC, 0.2% SDS and 0.1 × SSC) at room temperature with agitation. Slides were dried by centrifugation at 800 × *g* for 2 min and immediately scanned.

### Data acquisition and microarray data analysis

Hybridized microarrays were scanned using a GenePix 4000A dual-channel (635 nm and 532 nm) confocal laser scanner (Axon Instruments). The genes represented on the arrays were adjusted for background and normalized to internal controls using image analysis software (GenePixPro 4.0; Axon Instruments Inc.). Genes with fluorescent signal values below background were disregarded in all analyses. Statistical analysis was performed by Seralogix, LLC, Austin, TX, employing their computational tools termed the BioSignature Discovery System (BioSignatureDS) to determine significant gene modulations via a Bayesian *z*-score sliding window threshold technique and fold change. Normalizations against genomic DNA were performed as previously described [69]. The microarray data were deposited in the Gene Expression Omnibus (GEO) database at the National Center for Biotechnology Information (Accession #: GSE44688), including the ortholog gene references of *B. suis* attributed to the spot-IDs of the microarrays. For determination of significant differential gene expression, an absolute value for z-score cut-off of +/−1.96 was employed, which is equivalent to 95% confidence for the two-sided *t*-test with all genes differentially expressed having a plain fold change greater than the absolute value of 1.5. More detailed description of the computational techniques employed by BioSignatureDS was described in previous publications [76,77].

### RT-qPCR analysis

Expression of randomly selected genes from most of the different functional categories (n = 19) that we defined based on microarray analysis, and which were differentially expressed between *B. suis* wild-type and the *rsh* mutant, was analyzed by quantitative RT-PCR (RT-qPCR). 800 ng from the same RNA samples used for microarray hybridization were reverse-transcribed using a 6-mer random primers mix, as described earlier [75], and quantitative RT-PCR experiments were performed using the Light Cycler 480 with SYBR green chemistry to monitor and quantify the amplification rate (Roche). Primers (Sigma Genosys) were designed using Primer 3 Software (Additional file 3: Table S2) to produce an amplicon length between 150 and 250 bp. For each gene tested, the mean calculated threshold cycles (Ct) of the wild-type and the mutant were averaged and normalized to the Ct of a gene with constant expression identified in the transcriptomic analysis. The normalized Ct was used for calculating the fold change using the ΔΔC_t_ method [78]. Briefly, relative fold change (Δ*rsh* mutant/wild-type) = 2^-ΔΔCt^, where ΔCt (Gene of interest) = Ct (Gene of interest)-Ct (Reference gene of the same sample, BR1035) and ΔΔCt (Gene of interest) = ΔCt (Δ*rsh* mutant)-ΔCt (wild-type). BR1035, characterized by constant expression in both strains in minimal medium, was used as reference gene. For each primer pair, a negative control (water) and a RNA sample without reverse transcriptase (to determine genomic DNA contamination) were included as controls during cDNA quantification. Array data were considered valid if the fold change of each gene tested by RT-qPCR was log2(x) > 0.66 or log2(1/x) < −0.66 (representing a plain fold-change > 1.5) and consistent with microarray analysis.

### Nitrate reductase assay

To assess the amount of nitrite produced, the culture supernatants were assayed for nitrite accumulation by a spectrophotometric assay based on the Griess reaction [44]. *B. suis* wild-type and Δ*rsh* were washed in PBS, and incubated in GMM pH 7.0 containing 10 mM of NaNO_3_ for 6 h at 37°C under vigorous shaking for aeration. The nitrite concentration was measured with 100 μl of pure or diluted culture supernatants in 100 μl of Griess reagent containing 1% sulfamide in 70% acetic acid, and 0.1% N (naphthyl) ethylenediamine in 60% acetic acid. A pink color indicated nitrites in the supernatants, and OD was measured at 570 nm. Nitrite concentrations between 0 and 200 μM were used for a calibration curve.

### Measurement of *in vitro* sensitivity to superoxide production

A previously described procedure was used to follow up superoxide dismutase activity [52]. Briefly, mid-log phase cultures of *B. suis* wild-type, the Δ*rsh* mutant and the complemented mutant grown in Tryptic Soy broth were washed twice in phosphate-buffered saline (PBS) and adjusted to a density of approximately 10^6^ CFU/ml. Xanthine at the final concentration of 2 mM and 1 U/ml of xanthine oxidase were added to these cell suspensions along with 1,000 U/ml of bovine liver catalase to detoxify any H_2_O_2_ generated by spontaneous dismutation of the O_2_^-^ produced during the xanthine oxydase reaction. At specific time points after initiating the xanthine oxidase reaction, the number of surviving bacteria in the cell suspension was determined by 10-fold serial dilutions and plating on TS agar. Plates were incubated for 3 days at 37°C. The means from 3 independent platings of each bacterial cell suspension were calculated, and the data obtained were expressed as log10 CFU/ml at each sampling time, ± standard deviations.

### Construction of *rsh* and methionine mutants of *B. suis*

The *rsh* null mutant has been described previously [23]. The mutants in methionine biosynthesis genes (*metA*, *metZ*, *metH* and BR0793) contained a kanamycin resistance gene, replacing an internal portion of the target gene. For this purpose, 1239-, 1484-, 4045-, and 1451-bp fragments of *metA*, *metZ*, *metH* and BR0793, respectively, were amplified by PCR from the genomic DNA of *B. suis* with specific primers listed in Additional file 3: Table S2 (Sigma Genosys). These PCR fragments were inserted into pGEM-T Easy (Promega). The resulting plasmids were digested by *BssH*II, *Bsm*I*-Bam*HI, *Stu*I, and *Bsm*I*-Hind*III, respectively, to delete internal DNA fragments, and treated with T4-DNA-polymerase to obtain blunt-ended extremities. The deleted fragments were replaced by the kanamycin resistance gene excised from plasmid pUC4K using *Hinc*II. To generate *met* mutants, the resulting suicide plasmids were introduced into *B. suis* by electroporation. Kan^R^/Amp^S^ allelic exchange mutants were selected and validated by PCR. For complementation, the *metH* was excised from pGEM-T and subcloned into the *Apa*I-*Sac*I sites of pBBR1MCS. The obtained plasmid pBBR1MCS-*metH* was transformed into *ΔmetH*_Bs_. Complemented *ΔmetH* mutants were selected on the basis of their Kan^R^-Cm^R^ phenotype.

### Macrophage infection experiments with *Brucella* strains

Experiments were performed as described previously [79]. Briefly, murine J774A.1 macrophage-like cells were infected with early-stationary-phase *B. suis* strains for 45 min at a multiplicity of infection (MOI) of 20 bacteria per cell. Cells were washed twice with PBS and re-incubated in RPMI 1640 with 10% fetal calf serum and gentamicin at 30 μg/ml for at least 1 h to kill extracellular bacteria. After incubation of macrophages at 37°C and 5% CO_2_ for various time periods, cells were washed twice with PBS and lysed in 0.2% Triton X-100. The number of intracellular live bacteria was determined by plating serial dilutions on TS agar plates and incubation at 37°C for 3 days. All experiments were performed at least three times in triplicate.

## Competing interests

KD is an employee of Seralogix, LLC, and has an equity position in this company. Seralogix, LLC is a private business developing the software analysis and modeling software for commercial purposes which was utilized in the analysis of the data in this article.

The other authors declare that they have no competing interests.

## Authors’ contributions

NH, SOB, and AO carried out the experiments. NH, AO and SK conceived the study and contributed to the interpretation of the data. GA and KD developed the microarrays and performed and contributed to the microarray data normalization and processing. NH, SOB, AO and SK were involved in drafting the manuscript. All authors read and approved the final manuscript.

## Supplementary Material

Additional file 1: Table S1 Up-regulation (grey) and down-regulation (white) of Rsh-dependent genes in the *B. suis* wild-type, as determined by transcriptome analysis of the stringent response, in comparison to the *rsh*-mutant. Click here for file

Additional file 2: Figure S1 Comparison of microarray analysis to real-time PCR revealed 78% of true positives. Fold-change of differentially expressed genes is shown for normalized microarray data and RT-qPCR of 27 ORFs (out of 40 total; see also Figure 2) representing the different functional groups: (A) H- DNA/RNA metabolism; I- Energy metabolism; J- Fatty acid metabolism; K- Nitrogen metabolism; L- Protein metabolism; N- Regulation. (B) O: Stress and adaptation/chaperones/protein folding; P: Sugar metabolism; Q: Transport systems; R: Transposon function; S: Unknown function. Expression of 78% of all chosen ORFs (see also Figure 2) was consistent for both methods, with a fold-change superior to 1.5. For the remaining 22%, the fold-change was inferior to 1.5 or not consistent with microarray analysis. Relative differences of transcription levels between *B. suis* wild-type and the Δ*rsh* mutant were determined as 2^-ΔΔCt^ values, as described in Methods. Click here for file

Additional file 3: Table S2 Oligonucleotides used in this study. Click here for file
