# Supplementary material for: Global Rsh-dependent transcription profile of Brucella suis during stringent response unravels adaptation to nutrient starvation and cross-talk with other stress responses
Source: BMC Genomics. 2013 Jul 8;14:459. doi: 10.1186/1471-2164-14-459 (PMC3710219; doi:10.1186/1471-2164-14-459)
Supplement: Additional file 1: Table S1 — Up-regulation (grey) and down-regulation (white) of Rsh-dependent genes in the B. suis wild-type, as determined by transcriptome analysis of the stringent response, in comparison to the rsh-mutant. [file 1471-2164-14-459-S1.pdf]

Table S1. Up-regulation (grey) and down-regulation (white) of Rsh-dependent genes in the *B. suis* wild-type, as determined by transcriptome analysis of the stringent response.

Functional group	Gene ID	Relative Fold WT/Δ*rsh* (log2)	Annotated Function[a]
**Amino acid metabolism**			
	BRA0338	1.58	glutamate decarboxylase GadA, authentic point mutation
	BRA0340	1.33	glutaminase
	BR0793	0.88	O-acetylhomoserine aminocarboxypropyltransferase
	BR0679	-0.82	2-isopropylmalate synthase
	BR0232	-0.83	*soxG*: sarcosine oxidase, gamma subunit
	BR0646	-0.84	*dapA*: dihydrodipicolinate synthase
	BR1293	-0.89	branched-chain amino acid ABC transporter, periplasmic amino acid-binding protein, putative
	BR1932	-0.91	*dapF*: diaminopimelate epimerase
	BR1983	-0.93	*lysA*: diaminopimelate decarboxylase
	BRA0890	-0.93	*leuB*: 3-isopropylmalate dehydrogenase
	BR1988	-0.99	*tyrC*: prephenate dehydrogenase
	BR0020	-0.99	*ivd*: isovaleryl-CoA dehydrogenase
	BRA0932	-1.20	*hutU*: urocanate hydratase
	BR1140	-1.30	*trpD*: anthranilate phosphoribosyltransferase
	BR0614	-1.32	*glnE*: glutamate-ammonia-ligase adenylyltransferase
	BRA0486	-1.39	*metA*: homoserine O-succinyltransferase
**Cell division**			
	BRA0530	1.26	cell division protein FtsK, putative
	BR1895	1.04	cell division protein FtsK, putative
	BR0881	-1.06	*scpB*: segregation and condensation protein B
**Cell envelope**			
	BR0119	1.86	outer membrane protein, putative
	BR0783	1.75	capsule polysaccharide export protein, putative
	BR1930	1.62	*omp19*: lipoprotein Omp19
	BR0982	1.55	*wbdA*: glycosyl transferase, group 1 family protein
	BR0971	1.52	outer membrane protein, putative
	BRA0137	1.38	dolichyl-phosphate-mannose-protein mannosyltransferase family protein
	BR0433	1.34	transglycosylase-associated protein, putative
	BRA0423	1.25	*omp31-2*: outer membrane protein, 31 kDa
	BRA0542	1.24	NAD-dependent epimerase/dehydratase family protein
	BR1622	1.16	*omp31-1*: outer membrane protein Omp31
	BR0615	1.15	*lpsA*: hypothetical protein
	BR0915	1.03	*amiC*: N-acetylmuramoyl-L-alanine amidase, family 3
	BR0578	0.98	peptidoglycan-binding protein, putative

	BR0118	0.98	outer membrane protein, putative
	BR0511	0.92	*wbpL*: glycosyl transferase, group 4 family protein
	BR1205	0.90	lipoprotein, putative
	BR1133	-0.89	*kdsA*: 2-dehydro-3-deoxyphosphooctonate aldolase
	BRA0381	-0.90	membrane antigen, putative
	BR0909	-0.90	*dsbA*: outer membrane protein, putative
	BR0501	-1.01	lipoprotein, putative, authentic point mutation
	BR2135	-1.07	rare lipoprotein A family protein
	BRA0487	-1.13	glycosyl hydrolase family 25
	BR1435	-1.31	*murF*: UDP-N-acetylmuramoylalanyl-D-glutamyl-2,6-diaminopimelate-D-alanyl-D-alanyl ligase
	BR1204	-1.70	OmpA family protein
**Central intermediate metabolism**			
	BR1468	1.34	choloylglycine hydrolase family protein
	BR1993	-1.90	*ppa*: inorganic pyrophosphatase
**Chemotaxis & motility**			
	BRA1136	-0.96	*flaF*: flagellar protein FlaF, putative
	BRA0122	-0.86	*fliG*: flagellar motor switch protein FliG, authentic point mutation
**Cofactor & carrier biosynthesis**			
	BR1365	1.25	*cbiM*: cobalamin biosynthesis protein CbiM
	BRA0489	1.12	*bioA*: adenosylmethionine-8-amino-7-oxononanoate transaminase
	BR2066	1.10	*hemE*: uroporphyrinogen decarboxylase
	BR0330	1.08	*panB*: 3-methyl-2-oxobutanoate hydroxymethyltransferase
	BR1464	0.99	*pncA*: pyrazinamidase/nicotinamidase
	BR0683	-0.82	*pdxA*: 4-hydroxythreonine-4-phosphate dehydrogenase
	BR0866	-0.83	*cobS*: cobalamin synthase
	BR1142	-0.86	*moaC*: molybdenum cofactor biosynthesis protein C
	BR2131	-0.88	*gshB*: glutathione synthetase
	BR1299	-0.92	*cobK*: precorrin-6x reductase
	BR1289	-0.93	*cbiG*: protein/precorrin-3B C17-methyltransferase
	BR1124	-0.99	*lipA*: lipoyl synthase
	BRA0013	-1.19	*entA*: 2,3-dihydroxybenzoate-2,3-dehydrogenase
	BRA0015	-1.22	*entE*: 2,3-dihydroxybenzoate-AMP ligase
**Detoxification**			
	BRA0703	2.40	*sodC*: superoxide dismutase, Cu-Zn
**DNA/RNA metabolism**			
	BR1105	1.26	DNA-binding protein HU
	BRA0317	1.07	*nrdF*: ribonucleotide-diphosphate reductase beta subunit
	BRA0751	1.04	endoribonuclease L-PSP, putative
	BRA0599	0.96	*pyrB*: aspartate carbamoyltransferase catalytic subunit
	BR2183	0.96	*mutM*: formamidopyrimidine-DNA glycosylase
	BR1399	0.93	*thyA*: thymidylate synthase
	BR1804	0.90	*priA*: primosome assembly protein PriA

	BR0778	0.88	*himA*: integration host factor alpha subunit
	BRA0604	0.87	*topA*: DNA topoisomerase I
	BR0032	0.86	*recR*: recombination protein RecR
	BRA0841	-0.89	*hsdS*: type I restriction-modification system, S subunit
	BR0873	-0.97	*xth-1*: exodeoxyribonuclease III
	BR1283	-0.99	*radC*: DNA repair protein RadC
	BRA0840	-1.16	*hsdM*: type I restriction-modification system, M subunit
	BR0842	-1.20	*purC*: phosphoribosylaminoimidazole-succinocarboxamide synthase
	BR1249	-1.21	*nusG*: transcription antitermination protein NusG
**Energy metabolism**			
	BRA0297	1.38	*narJ*: respiratory nitrate reductase, delta subunit
	BRA0571	1.13	*qor*: quinone oxidoreductase
	BRA1014	1.08	aldehyde dehydrogenase family protein
	BR1061	1.05	alcohol dehydrogenase, zinc-containing
	BR0202	1.05	aldehyde dehydrogenase family protein
	BRA0168	1.04	citrate lyase, beta subunit, putative
	BRA1171	0.99	aldehyde dehydrogenase family protein
	BR0363	0.94	*ccoN*: cytochrome *c* oxidase, *cbb3*-type, subunit I
	BR0360	0.90	*ccoP*: cytochrome *c* oxidase, *cbb3*-type, subunit III
	BR0203	0.87	alcohol dehydrogenase, zinc-containing
	BRA0299	0.86	*narG*: respiratory nitrate reductase, alpha subunit
	BR1801	-0.82	*atpA:* ATP synthase subunit A
	BR1970	-0.82	*etfA*: electron transfer flavoprotein, alpha subunit
	BR1901	-0.84	*sdhB*: succinate dehydrogenase catalytic subunit
	BRA0893	-1.05	*trx-2*: thioredoxin
	BR0381	-0.89	ATP synthase protein I, putative
	BRA1058	-0.89	aldehyde dehydrogenase family protein
	BRA0386	-0.91	acetate kinase
	BR0384	-0.93	ATP synthase subunit B
	BR1903	-0.93	succinate dehydrogenase, hydrophobic membrane anchor protein, putative
	BR0382	-1.30	*atpB*: ATP synthase subunit A
	BR0852	-1.48	alcohol dehydrogenase, zinc-containing
**Fatty acid metabolism**			
	BR1510	0.99	long-chain acyl-CoA thioester hydrolase, putative
	BR0289	0.86	*fadD*: acyl-CoA synthase
	BR1994	-0.84	1-acyl-sn-glycerol-3-phosphate acyltransferase, putative
	BR0907	-0.86	*accB*: acetyl-CoA carboxylase
	BR2107	-1.07	*accD*: acetyl-CoA carboxylase beta subunit
	BR0698	-1.31	*pgsA*: CDP-diacylglycerol-glycerol-3-phosphate 3-phosphatidyltransferase
**Nitrogen metabolism**			
	BR1358	1.86	*ureC*-2: urease, alpha subunit
	BR1360	1.70	*ureF*: urease accessory protein UreF, putative
	BR1357	1.61	*ureB*-2: urease, beta subunit

	BR1361	1.46	*ureG*-2: urease accessory protein UreG
	BR1356	1.22	*ureA*-2: urease, gamma subunit
	BR1359	1.11	*ureE*: urease accessory protein UreE, putative
	BR0270	-0.96	*ureC*-1: urease, alpha subunit
	BR0271	-1.21	*ureE*: urease accessory protein UreE, putative
	BR0273	-1.90	*ureG*-1: urease accessory protein UreG
	BR0267	-2.33	*ureD*-1: urease accessory protein UreD
	BR0269	-3.06	*ureB*-1: urease, beta subunit
**Protein metabolism**			
	BR1197	2.35	ThiJ/PfpI family protein
	BR1861	1.38	peptidase, M23/M37 family
	BRA0289	1.30	protease, putative
	BR1915	0.87	*rimM*: 16S rRNA-processing protein
	BR1222	-0.83	*rplX*: 50S ribosomal protein L24
	BR0755	-0.83	arginyl-tRNA-protein transferase
	BR0455	-0.87	*rpsF*: 30S ribosomal protein S6
	BR1223	-0.87	*rplN*: 50S ribosomal protein L14
	BR2015	-0.87	*rpmB*: 50S ribosomal protein L28
	BR2117	-0.88	*infC*: translation initiation factor IF-3
	BR0677	-0.88	*cysS*: cysteinyl-tRNA synthetase
	BR1227	-0.89	*rpsC*: 30S ribosomal protein S3
	BR0404	-0.89	*glyS*: glycyl-tRNA synthetase beta subunit
	BR1251	-0.90	*tuf*: elongation factor Tu
	BR1535	-0.91	50S ribosomal protein L25
	BR1219	-0.95	*rpsH*: 30S ribosomal protein S8
	BR1228	-0.95	*rplV*: 50S ribosomal protein L22
	BR1215	-0.95	*rpmD*: 50S ribosomal protein L30
	BR1907	-0.96	*rplS*: 50S ribosomal protein L19
	BR1231	-0.96	*rplW*: 50S ribosomal protein L23
	BR0454	-0.99	*rpsR*: 30S ribosomal protein S18
	BR1245	-0.99	*rplL*: 50S ribosomal protein L7/L12
	BR0822	-0.99	*proS*: prolyl-tRNA synthetase
	BR1232	-1.07	*rplD*: 50S ribosomal protein L4
	BR1162	-1.10	*rpsB*: 30S ribosomal protein S2
	BR1161	-1.10	*tsf*: elongation factor Ts
	BR0314	-1.08	*pip*: proline iminopeptidase
	BR1233	-1.15	*rplC*: 50S ribosomal protein L3
	BR1216	-1.17	*rpsE*: 30S ribosomal protein S5
	BR1214	-1.19	*rplO*: ribosomal protein L15
	BR0027	-1.20	*rpsA*: 30S ribosomal protein S1
	BR1230	-1.22	*rplB*: 50S ribosomal protein L2
	BR1220	-1.25	*rpsN*: 30S ribosomal protein S14
	BR1218	-1.27	*rplF*: 50S ribosomal protein L6

	BR1229	-1.28	*rpsS*: 30S ribosomal protein S19
	BR1196	-1.29	RNA methyltransferase, TrmH family, group 1
	BR1849	-1.31	*rpmA*: 50S ribosomal protein L27
	BR1236	-1.38	*fusA*: elongation factor EF-2
	BR1234	-1.39	*rpsJ*: 30S ribosomal protein S10
	BR1237	-1.57	*rpsG*: 30S ribosomal protein S7
	BR0830	-1.58	*rpsD*: 30S ribosomal protein S4
	BR1238	-1.63	*rpsL*: 30S ribosomal protein S12
**Protein modification & repair**			
	BR0905	-0.82	*aat*: leucyl/phenylalanyl-tRNA-protein transferase
**Regulation**			
	BR1100	1.92	transcriptional regulator, GntR family
	BRA0744	1.56	transcriptional regulator, RpiR family
	BR1613	1.54	transcriptional regulator, Cro/CI family
	BR0201	1.21	transcriptional regulator, Fis family, authentic frameshift
	BR0569	1.20	transcriptional regulator, MucR
	BR2017	1.17	transcriptional regulator, MerR family
	BRA1074	1.06	transcriptional regulator, GntR family
	BR1086	1.03	transcriptional regulator, LysR family
	BRA0927	1.00	*hutC*: histidine utilization repressor
	BR0556	0.96	transcription elongation factor GreA domain protein
	BR0102	0.90	response regulator, putative
	BRA0820	0.89	transcriptional regulator, MerR family
	BRA0610	0.87	response regulator/GGDEF domain protein
	BR1187	-0.86	transcriptional regulator, Cro/CI family
	BRA0354	-0.98	*oxyR*: transcriptional regulator OxyR
	BR1115	-1.02	*ntrX*: nitrogen regulation protein NtrX
	BR0872	-1.08	*exoR*: exopolysacchride production negative regulator
**Stress & adaptation**			
	BR0171	2.08	*grpE*: heat shock protein GrpE
	BR1047	1.40	Universal stress protein UspA and related nucleotide-binding proteins
	BR1514	1.19	cold-shock family protein Csp
	BRA0341	1.02	*hdeA*: hdeA protein
	BR0318	1.01	*mscL*: large conductance mechanosensitive channel protein
	BR2126	0.99	*dnaJ*: chaperone protein DnaJ
	BRA0295	0.95	peptidyl-prolyl cis-trans isomerase, putative
	BRA0184	-0.84	ATP phosphoribosyltransferase regulatory subunit
	BRA0188	-0.85	*groEL*: chaperonin GroEL
	BR1943	-0.94	peptidyl-prolyl cis-trans isomerase
	BRA0195	-1.07	*groES*: co-chaperonin GroES
**Sugar metabolism**			
	BR1729	1.48	*pgk*: phosphoglycerate kinase
	BR2042	-0.93	L-sorbose dehydrogenase, FAD dependent, putative

	BR0879	-1.02	glycosyl hydrolase, family 3
	BRA0865	-1.05	*eryB*: erythritol phosphate dehydrogenase
	BRA0543	-1.10	*galE*-2: UDP-glucose 4-epimerase
	BR2042	-1.31	L-sorbose dehydrogenase, FAD dependent, putative
	BRA0871	-1.86	*fbaA*: fructose-bisphosphate aldolase
**Transport systems**			
	BRA0408	1.98	peptide ABC transporter, permease protein
	BR1349	1.86	ABC transporter, permease protein, putative
	BRA0651	1.56	branched-chain amino acid ABC transporter, ATP-binding protein
	BR0639	1.47	*omp2b*: porin Omp2a
	BR1350	1.44	ABC transporter, ATP binding/permease protein
	BRA0300	1.42	*narK*: nitrite extrusion protein
	BR1959	1.23	amino acid ABC transporter, ATP-binding protein
	BRA0534	1.18	ABC transporter, ATP-binding protein
	BRA0334	1.16	hydrophobe/amphiphile efflux-1 family protein
	BRA0115	1.16	ABC transporter, periplasmic substrate-binding protein
	BR0225	1.07	ABC transporter, periplasmic substrate-binding protein, putative
	BRA1196	1.05	branched-chain amino acid ABC transporter, ATP-binding protein
	BRA0565	1.05	*bfr*: bacterioferritin
	BRA0334	1.04	hydrophobe/amphiphile efflux-1 family protein
	BR1060	1.04	HlyD family secretion protein
	BR1325	1.03	potassium efflux system family protein
	BRA1012	1.01	*dppA*: ABC transporter, periplasmic substrate-binding protein
	BR0371	1.01	membrane protein, putative, authentic point mutation
	BRA0503	0.98	monovalent cation/proton antiporter, MnhE/PhaE subunit
	BRA0749	0.97	sugar ABC transporter, permease protein, putative
	BR0637	0.96	*omp2a*: porin Omp2a
	BRA0335	0.95	HlyD family secretion protein
	BRA0065	0.95	*virB5*: type IV secretion system protein VirB5
	BRA0657	0.94	*ugpE*: glycerol-3-phosphate ABC transporter, permease protein
	BR1059	0.94	drug resistance transporter, EmrB/QacA family
	BRA1081	0.90	peptide ABC transporter, permease protein
	BR1568	0.87	cation efflux family protein
	BR1490	0.86	transporter, putative
	BR0222	0.86	ABC transporter, permease protein
	BR0291	-0.82	HlyD family secretion protein
	BR0882	-0.87	*tatA*: twin argininte translocase protein A
	BR1699	-0.90	TonB system transport protein, ExbD/TolR family
	BRA0948	-0.90	amino acid ABC transporter, periplasmic amino acid-binding protein
	BRA0115	-0.90	drug resistance transporter, Bcr/CflA family
	BRA0993	-0.90	*rbsC*-4: ribose ABC transporter, permease protein
	BRA0675	-0.91	iron compound ABC transporter, periplasmic iron compound-binding protein

	BRA0953	-0.92	branched-chain amino acid ABC transporter, periplasmic amino acid-binding protein, putative
	BR2141	-0.92	*pstB*: phosphate ABC transporter, ATP-binding protein
	BR1137	-0.97	protein-export membrane protein
	BRA0518	-0.99	sugar ABC transporter, permease protein
	BR0743	-0.99	amino acid ABC transporter, permease protein
	BR1269	-0.99	hypothetical protein BR1269
	BRA1183	-1.07	sugar ABC transporter, ATP-binding protein, putative
	BRA0090	-1.09	*modC*: molybdenum ABC transporter, ATP-binding protein
	BRA0740	-1.14	glycine betaine/L-proline ABC transporter, ATP-binding protein
	BR1490	-1.16	transporter, putative
	BR2055	-1.20	branched-chain amino acid ABC transporter, permease protein
	BR1700	-1.26	*tolQ*: tolQ protein
	BRA0519	-2.16	sugar ABC transporter, permease protein
**Transposon function**			
	BRA0557	1.87	IS66 family element, orf2, putative
	BR0523	1.75	ISBm1, transposase orfA
	BRA0559	1.55	ISBm1, transposase orfA
	BRA0556	1.32	IS66 family element, orf3, putative
	BR0533	1.12	ISBm1, transposase orfA
	BR1673	1.03	IS711, transposase orfB
	BRA0554	0.99	ISBm1, transposase orfB
	BR0513	0.99	transposase, degenerate
	BRA0552	0.93	IS711, transposase orfA
**Unknown function**			
	BR0629	2.41	hypothetical protein
	BRA0325	1.84	hypothetical protein
	BRA0332	1.83	hypothetical protein
	BRA0331	1.78	hypothetical protein
	BR1355	1.62	hypothetical protein
	BRA0564	1.61	hypothetical protein
	BRA0665	1.59	hypothetical protein
	BR0286	1.52	hypothetical protein
	BR1593	1.51	hypothetical protein
	BR1809	1.42	hypothetical protein
	BRA0413	1.42	hypothetical protein
	BR0727	1.37	hypothetical protein
	BRA0716	1.32	*iolB* protein, authentic frameshift
	BR0962	1.32	hypothetical protein
	BR1366	1.31	hypothetical protein
	BRA0291	1.30	hypothetical protein
	BR0983	1.27	hypothetical protein
	BRA1054	1.26	nitroreductase family protein

	BR2063	1.26	hypothetical protein
	BRA0625	1.25	hypothetical protein
	BR1353	1.23	hypothetical protein
	BRA0818	1.20	*crcB* family protein
	BR1856	1.19	hypothetical protein
	BR0207	1.19	hypothetical protein
	BR1496	1.17	hypothetical protein
	BRA0196	1.17	hypothetical protein
	BR0730	1.16	transposition protein, putative
	BR0969	1.16	hypothetical protein
	BRA0141	1.14	SyrB family protein, putative
	BR1046	1.13	phosphoribosyltransferase family protein
	BR0892	1.12	hypothetical protein
	BRA0575	1.12	hypothetical protein
	BRA0635	1.11	twin-arginine translocation signal domain protein
	BR0734	1.10	hypothetical protein
	BR1206	1.10	ATPase, AAA family
	BR1841	1.10	iojap-related protein
	BR2136	1.08	hypothetical protein
	BRA0264	1.08	hypothetical protein
	BRA0168	1.07	hypothetical protein
	BR0084	1.06	hypothetical protein
	BR0049	1.04	hypothetical protein
	BRA0613	1.04	hypothetical protein
	BR0165	1.04	hypothetical protein
	BR2133	1.03	hypothetical protein
	BRA0978	1.03	conserved hypothetical protein, authentic frameshift
	BRA1062	1.01	hypothetical protein
	BRA1026	1.00	hydrolase, haloacid dehalogenase-like family
	BRA0717	0.99	inositol monophosphatase family protein
	BR1626	0.99	hypothetical protein
	BR0226	0.99	hypothetical protein
	BRA0343	0.99	hypothetical protein
	BR0785	0.99	hypothetical protein
	BR0369	0.99	hypothetical protein
	BR1411	0.98	hypothetical protein
	BR1518	0.97	hypothetical protein
	BR1024	0.96	hypothetical protein
	BR1419	0.95	hypothetical protein
	BR0951	0.95	glutathione S-transferase domain protein
	BRA1037	0.94	hypothetical protein
	BRA0255	0.94	hypothetical protein
	BR1822	0.94	LemA protein, putative

	BRA0596	0.94	metal-dependent hydrolase
	BRA0824	0.93	hypothetical protein
	BR0517	0.91	formyltransferase, putative
	BRA0478	0.90	hypothetical protein
	BR0571	0.90	hypothetical protein
	BRA1061	0.90	hypothetical protein
	BRA0787	0.90	hypothetical protein
	BRA0606	0.90	hypothetical protein
	BRA0397	0.89	shikimate dehydrogenase family protein
	BR1054	0.89	hypothetical protein
	BRA0141	0.89	hypothetical protein
	BR2012	0.88	hypothetical protein
	BR1968	0.88	hypothetical protein
	BR1750	0.88	hypothetical protein
	BR0687	0.88	hypothetical protein
	BR0740	0.88	PAP2 family protein
	BR0180	0.88	hypothetical protein
	BR2007	-0.82	hypothetical protein
	BR1273	-0.83	*glpX*: glpX protein
	BR1291	-0.85	phospholipase/carboxylesterase family protein
	BRA0318	-0.86	hypothetical protein
	BRA1032	-0.86	hypothetical protein
	BRA0839	-0.87	hypothetical protein
	BR0140	-0.87	NifU-related protein
	BR1847	-0.87	acetyltransferase, GNAT family
	BR1921	-0.88	hypothetical protein
	BR0818	-0.89	glyoxalase family protein
	BR0746	-0.89	monooxygenase
	BR1164	-0.90	hypothetical protein
	BR1150	-0.90	conserved hypothetical protein
	BR0799	-0.90	hypothetical protein
	BR1378	-0.91	aminotransferase, class I
	BRA0879	-0.91	glyoxalase family protein
	BR1025	-0.92	hypothetical protein
	BR1641	-0.93	hypothetical protein
	BR0327	-0.96	hypothetical protein
	BRA0836	-0.96	hypothetical protein
	BRA0729	-0.98	pyridine nucleotide-disulphide oxidoreductase family protein
	BR1290	-0.99	hypothetical protein
	BR1113	-0.99	hypothetical protein
	BR0335	-1.00	hypothetical protein
	BR2008	-1.01	hypothetical protein
	BRA1201	-1.01	hypothetical protein

	BR0506	-1.01	BioY family protein
	BR1443	-1.04	transglycosylase SLT domain protein
	BR1752	-1.04	hypothetical protein
	BR0964	-1.05	hypothetical protein
	BR1327	-1.06	hypothetical protein
	BR1275	-1.06	hypothetical protein
	BRA0861	-1.06	hypothetical protein
	BR2098	-1.06	hypothetical protein
	BR0391	-1.08	hypothetical protein
	BR0838	-1.09	lipoprotein, putative
	BR1011	-1.11	hypothetical protein
	BR1136	-1.18	hypothetical protein
	BR1574	-1.22	hypothetical protein
	BR1516	-1.24	hypothetical protein
	BRA0873	-1.25	hypothetical protein
	BRA0382	-1.28	hypothetical protein
	BRA0759	-1.36	hypothetical protein
	BRA0476	-1.42	hypothetical protein
	BRA0875	-1.52	GTP-binding protein TypA
	BRA0438	-1.58	methyltransferase, putative
	BR0389	-1.71	hypothetical protein
	BR0391	-1.71	hypothetical protein
	BR2041	-1.82	hypothetical protein

[a]Annotations according to the "JCVI Comprehensive Microbial Resource" (J. Craig Venter Institute; http://cmr.jcvi.org/cgi-bin/CMR/CmrHomePage.cgi).
