# Supplementary figures and images for: Global Rsh-dependent transcription profile of Brucella suis during stringent response unravels adaptation to nutrient starvation and cross-talk with other stress responses
Source: BMC Genomics. 2013 Jul 8;14:459. doi: 10.1186/1471-2164-14-459 (PMC3710219; doi:10.1186/1471-2164-14-459)

A

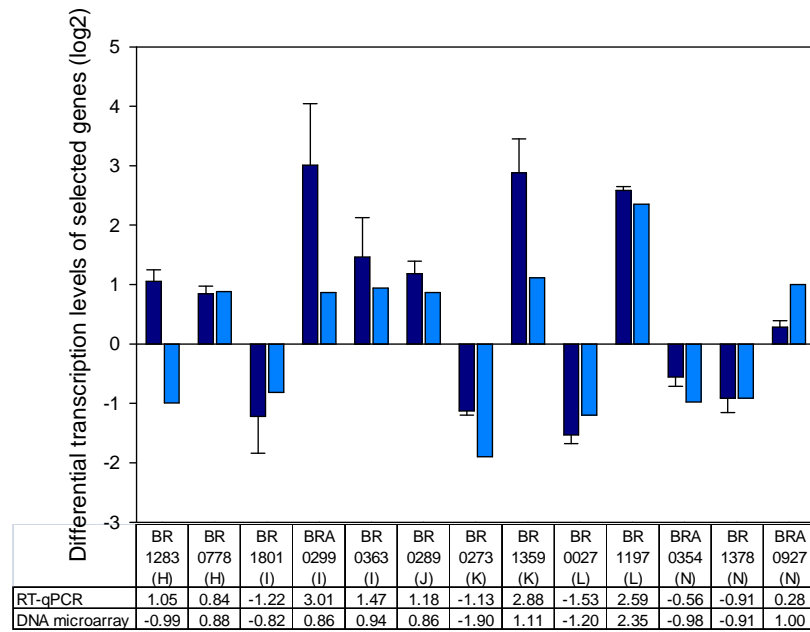

B

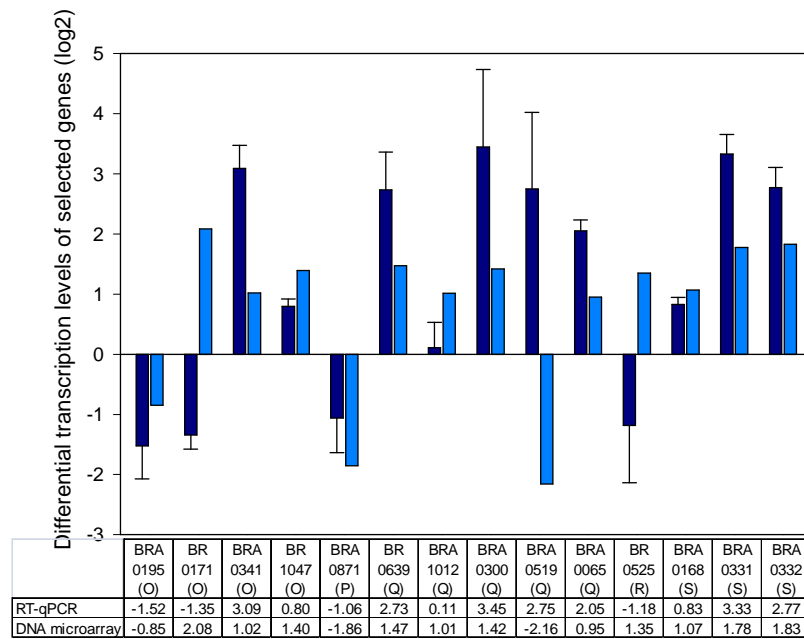

Figure S1

Supplement: Additional file 2: Figure S1 — Comparison of microarray analysis to real-time PCR revealed 78% of true positives. Fold-change of differentially expressed genes is shown for normalized microarray data and RT-qPCR of 27 ORFs (out of 40 total; see also Figure 2) representing the different functional groups: (A) H- DNA/RNA metabolism; I- Energy metabolism; J- Fatty acid metabolism; K- Nitrogen metabolism; L- Protein metabolism; N- Regulation. (B) O: Stress and adaptation/chaperones/protein folding; P: Sugar metabolism; Q: Transport systems; R: Transposon function; S: Unknown function. Expression of 78% of all chosen ORFs (see also Figure 2) was consistent for both methods, with a fold-change superior to 1.5. For the remaining 22%, the fold-change was inferior to 1.5 or not consistent with microarray analysis. Relative differences of transcription levels between B. suis wild-type and the Δrsh mutant were determined as 2-ΔΔCt values, as described in Methods. [file 1471-2164-14-459-S2.pdf]
