# Supplementary material for: Global Rsh-dependent transcription profile of Brucella suis during stringent response unravels adaptation to nutrient starvation and cross-talk with other stress responses
Source: BMC Genomics. 2013 Jul 8;14:459. doi: 10.1186/1471-2164-14-459 (PMC3710219; doi:10.1186/1471-2164-14-459)
Supplement: Additional file 3: Table S2 — Oligonucleotides used in this study. [file 1471-2164-14-459-S3.pdf]

Table S2. Oligonucleotides used in this study.

Designation	Sequence (5'- 3')	Designation	Sequence (5'- 3')
BR0118-F	TTACCAGCACCAGCGCATATG	BR2042-F	AGCACATGAAGAACCGTGTGC
BR0118-R	TGGTGCGGTTTTCAAGCTTGG	BR2042-R	GTCGTTGAAACGCTGATTGTC
BR0525-F	ACTACGAGATCGGCTACTTCC	BRA0299-F	GTCTGCATGTCCTTCTACGAC
BR0525-R	TTGTGGCCTTGCGATAGATAC	BRA0299-R	AGCATGAGGAAACCGGCATTG
BR0614-F	TTGTGGCTGGATCGTTCTTGG	BRA0300-F	GTTGCATTTGGCTGGCATACG
BR0614-R	GACACGGAAGACGTAACCATC	BRA0300-R	GACCTGAATGTTCTTGAGTGG
BR0982-F	AAGTTCCGTAAGAGACGGACG	BRA0331-F	GTGCGTTATCGAATCTTGAGG
BR0982-R	AGTCACTACAGCAATTGCAGC	BRA0331-R	ACACGGGCAATCTCATTACAG
BR0363-F	CTCAACCTCCAGCCCTACTTC	BRA0332-F	TGCAGATGGTTCTCTTGCTGG
BR0363-R	CCCAGAATACGAACCAGGCGAGA	BRA0332-R	CGTGGTTTCTCTGGGAATTCC
BR1197-F	CAACCGCCATTCACGACTTTG	BRA0338-F	TCAGAATCTGGCGACCTTCTG
BR1197-R	TCATCGAGGCGCAGATATTCG	BRA0338-R	TGACACAGCGCAGATCAATGG
BR1359-F	CCAAGATCGATTGGCTTGTGC	BRA0341-F	AAGGTGAGTGAACTCACCTGC
BR1359-R	CACTTCCTTGAGATGGACGTG	BRA0341-R	TACCATCAACGTCGATGACGG
BR1930-F	GCACGGTGCAGAAAGGCAATC	BRA0519-F	GCTTTAGCCTCATCGGTTATG
BR1930-R	TAGCCCTGGCCATATTTGGTC	BRA0519-R	ATGCCGATTGCCAGATAGAGC
BR1993-F	TCGACCGTTTCCTTTACACAC	BRA0530-F	CCTTGCAGATGATATCGCTCG
BR1993-R	CGAATTGTCTTCCATGACCAG	BRA0530-R	AGGCGATAATTGGACGACTCG
BR0778-F	CTGTGACGCTATCGTCAATGG	BRA0703-F	AGTTGGCACCGTGGTCATTTC
BR0778-R	TGACGCTTCTGATGTTCCTGC	BRA0703-R	GTGATGGGTATTACCCGGATC
BR0289-F	ACATCGTCAATCTGGTCGTGC	BRA0927-F	TGCATCAGCGTATTCTGGACG
BR0289-R	ATCCACAGGTTCATCTGCTCG	BRA0927-R	TTCACGGTCATGCGTGAACAG
BR1047-F	ACGGCTGAAGCATGTCTTCG	BR1801-F	ATGCTCTCGGCAATCCGATTG
BR1047-R	GCGGTTTCGTCATTGGTTGC	BR1801-R	GCTTCTGGTTGAGGAAGGTG
BR0615-F	CTGATTTCGACGTCGTGAAGC	BR1378-F	GATATCATTTCCTTCGCAGGC
BR0615-R	CAGTTCCATGGTCGATAGTGC	BR1378-R	ATCTTGGCGAGTTCGCCAAC
BR0273-F	AGACCACACTGACCGAAAAGC	BRA1012-F	TACAGGTTCAAGCTGCGTCAG
BR0273-R	ACGGTCTTCCGACAAGGCTTG	BRA1012-R	TGAACAGGAAATTCGAGGTCG
BR0171-F	ACGAAACCGCAAACCGTATCG	BR1983-F	GCAAGTCGGAGAACAAGTTCG
BR0171-R	AACCGAAAGCATGTCACGAGC	BR1983-R	GCAATTCCTTCACCAGTTCC
BRA0168-F	ATCCTTGATCTTGAGGATGCC	BR0027-F	GCGACGAAGTGGAAGTTTACG
BRA0168-R	AGAGCAATCAGCGGCAAGTCG	BR0027-R	CCACCCTTGACCTGATTGAAG
BRA0354-F	TGTTTCACAACCAGCACTCTCC	BR0305-F	TGACGCAAGGCTTCCTTTACC
BRA0354-R	TTGTGCGGCTATTTCCTCCAG	BR0305-R	CCGACCACCGACATGTTTGAA
BRA0557-F	TTCAGGAAGGGCATCAATGGG	BRA0486-F	GCCTTCTCAATCTGATGCCCA
BRA0557-R	CCAAACGACCATCTTCAGACG	BRA0486-R	CGACCACATGCTCTCCTTCTA
BRA0871-F	ACAACATGGAACAGGCACTGG	BR1450-F	GAATTCTTCCCTCCCAAGACG
BRA0871-R	TTGCCATGGTCGAGATGCACG	BR1450-R	GCACATCTGACCTGCGTGGAT
BR0119-F	GTTGTTGTTGCTCCGACCTTC	*met-H* For	CGGTGACTGTCCAAAACACG
BR0119-R	GAAACGTCGTTATAGGTCACG	*met-H* Rev	TCCACCGCAAAGCTTGAAGC
BRA0195-F	CTCGCTGACGCTGTTAAGGTC	Kan^R For	GGATTCAGTCGTCACTCATGG
BRA0195-R	AGTATCGTTGGTCTTGGAAGC	Kan^R Rev	CCATGAGTGACGACTGAATCC
BR0639-F	CGCTGGCTACTTCTACATTCC	BR1035-F	TCTTCTACGACATGCCAAAGC
BR0639-R	TGCCGAGTTCGGTTTCCGAAC	BR1035-R	AAAGCCCGGTTTCTTTCATCG
BR0793-F	TTGCGATTCCTGCATGTGACG	BR1871-F	TCAGGTTGCCGTGGTCGTTTC
BR0793-R	CAATGACGAGACATGCTGCGA	BR1871-R	TGAAGCGCAATTGCCAGAAGG
metA-F	TGCCAGAACACTATTGCCAGC	BR1274-F	CGTATTCTGCGTGACAAGTCC
metA-R	CGTCTTCCGATCCATGGAAGA	BR1274-R	CAGATTTGGCAAGTTCCACCG
metZ-F	ACCTGTTCCGTGGTGATTTGG		
metZ-R	CGTTGACGTCACCGCAACTTT		
